# Association of Genetic Variants of *ABCC8* Gene With Type 2 Diabetes Mellitus in the Mongolian Population

**DOI:** 10.1155/ije/5514592

**Published:** 2026-10-07

**Authors:** Nyamjav Byambasuren, Erkhembayar Shinebaatar, Solongo Enkhjargal, Purevdulam Erdenetsog, Munkhtsetseg Janlav, Buyankhuu Tuvshintur

**Affiliations:** ^1^ Anesthesiology and Intensive Care Unit State Secondary Central Hospital Ulaanbaatar Mongolia; ^2^ Department of Biochemistry School of Biomedicine Mongolian National University of Medical Sciences Ulaanbaatar Mongolia mnums.edu.mn

**Keywords:** *ABCC8* gene, *rs1801261* polymorphism, Sur1, Type 2 diabetes

## Abstract

**Introduction:**

Type 2 diabetes constitutes 90 percent of all diabetes cases, and some polymorphism in the *ABCC8* gene, which encodes the ATP‐sensitive potassium channel Sur1 protein involved in pancreatic beta cell insulin release, are associated with Type 2 diabetes. Sulfonylurea is used to treat diabetes caused by this metabolic disorder. Recent studies indicate that the rs1799854 and rs1801261 polymorphisms of the *ABCC8* gene may be associated with the development of Type 2 diabetes.

**Materials and Methods:**

This study was designed as a case–control study. Group 89 included individuals with Type 2 diabetes mellitus (T2DM), while the control group included 89 individuals determined healthy based on specific criteria. DNA was extracted from the blood, and the rs1799854 and rs1802161 polymorphisms of the *ABCC8* gene were analyzed using the RELP‐PCR method.

**Results:**

Our study’s results showed significant differences in BMI, glucose, and glycated hemoglobin levels between the case and control groups. Individuals with Type 2 diabetes showed higher frequencies of the TT genotype and the *T* allele of the rs1799854 polymorphism in the *ABCC8* gene (*p* < 0.05). The high frequency of the mutant *T* allele and TT genotype in the case group suggests that individuals carrying these genetic variations may have an increased susceptibility to Type 2 diabetes. Individuals with and without T2DM exhibited similar frequencies of the *T* allele and TT genotype of the rs1801261 polymorphism in the *ABCC8* gene (*p* > 0.05). Moreover, no statistically significant differences were detected between the case and control groups in those carrying risk alleles versus those not (*p* > 0.05).

**Conclusion:**

The rs1801261 polymorphisms of the *ABCC8* gene were not associated with Type 2 diabetes. However, findings indicated that the *ABCC8* 1,799,854 polymorphism may affect susceptibility to T2DM in this study.

## 1. Introduction

Diabetes is a metabolic disorder characterized by increased blood glucose levels due to decreased insulin secretion and impaired insulin action, resulting in frequent urination and excessive drinking [1]. It is a disorder in which blood glucose levels rise because the body’s cells become resistant to the hormone insulin. The International Diabetes Federation’s 2021 report indicates that 537 million individuals globally suffer from diabetes, with projections indicating a rise to 783 million by 2045 [1]. In our country, as of 2020, diabetes accounted for 41.2% of endocrine and metabolic diseases, and 17.4% of the population had altered fasting blood glucose levels. The genes encoding ATP‐dependent potassium channels on the membrane of pancreatic *β* cells are *KCNJ11* and *ABCC8*, and insulin secretion is impaired due to the function of this protein [2]. When blood glucose increases, ATP increases in the cytoplasm of cells as a result of glucose oxidation. ATP binds to the potassium channel, closing it and causing the cell membrane to depolarize; opening the calcium channel increases the amount of calcium entering the cell. Calcium entering the cell cytoplasm affects the formation of insulin granules, which releases insulin from the cell. Consequently, the release of insulin leads to a decrease in blood glucose levels [3, 4]. Many studies have suggested that some polymorphisms of the *ABCC8* gene, which encodes the potassium channel Sur1 protein, contribute to the development of diabetes. Studies conducted in several countries have suggested that the rs1799854 and rs1801261 polymorphisms of the *ABCC8* gene may contribute to the development of Type 2 diabetes [2, 5–9]. In our country, there has not yet been a study on the relationship between Type 2 diabetes and *ABCC8* gene polymorphisms, which is the reason for our study.

## 2. Materials and Methods

### 2.1. Study Subjects

This study was designed as a case–control study. The American Diabetes Association criteria (a fasting plasma glucose of more than or equal 126 mg/dL, or a plasma glucose of more than or equal 200 mg/dL after 2 h of oral glucose (1.75 g/kg), or an HbA1c of more than or equal 6.5%) guided the selection of 89 individuals with Type 2 diabetes mellitus (T2DM) in the case group. In the control group, 89 individuals met specific criteria: they appeared healthy, with a fasting plasma glucose of less than 100 mg/dL, an HbA1c of less than 5.7%, and no family history of T2DM. The study assumed an equal size for cases and controls, maintaining a 1:1 ratio.

### 2.2. Inclusion Criteria

• Individuals who have agreed to participate in the study or have read the informed consent and agreed to participate in the study
• Are ready to undergo the examination (fasting without food or drink for 8 h)

### 2.3. Exclusion Criteria

• Pregnant individuals with Type 1 diabetes
• Under 20 years of age
• Not following the precautions before the test
• Laboratory and instrumental tests have confirmed the diagnosis of a specific disease (heart disease, cancer, systemic diseases, endocrine diseases, and impaired kidney and liver function)

### 2.4. Biochemical Parameters

After obtaining consent from all participants, data were collected using a questionnaire, and fasting blood samples were collected in EDTA‐containing tubes. The measurements for glucose, total cholesterol, triglycerides, HDL, and LDL were performed using a fully automated analyzer called “Respons‐910,” manufactured by the German company Diasys. Additionally, blood glycated hemoglobin (HbA1c) was measured from the EDTA samples using a semiautomated SD A1cCare analyzer, with samples stored at 4°C for up to 7 days.

### 2.5. Genotyping of SNPs

DNA was extracted according to the accompanying protocol using the “Quick Prep Universal Genome DNA Kit” (Cat.№. A0204) by Hualikexi, China. *ABCC8* gene rs1799854 and rs1802161 polymorphisms were defined using a unique primer (Forward 5′‐TTGGGTGCATCTGTCTGTCTGTCTTT‐3′ and ‘Reverse 5′‐ AGCCCACCTGCCCCACGAT ‐3′) and (Forward 5′‐AGCACCCTGGAGGGAGTTGA‐3′ and ‘Reverse 5′‐ TCCCTCTTAACTGGGTCCTCC‐3′) to amplify the target sequence defined by the restriction fragment length polymorphism‐PCR method. Amplified DNA was digested using Pst1 and BsiE1 restriction enzyme (New England Biolabs, Beverly, MA, USA) at 37°C for 1 hour. The digested products were determined by 3% gel electrophoresis with ethidium bromide.

### 2.6. Statistical Analysis

Data collected were processed using SPSS 23.0. The chi‐square test was employed to assess whether the genotype distributions adhered to Hardy–Weinberg equilibrium (HWE). The frequencies of genotypes and alleles were compared between the two groups using the chi‐square test. The odds ratios were calculated using a logistic regression model. *p* value  <  0.05 was considered statistically significant.

### 2.7. Ethics

The study protocol was approved by the Ethical Committee of the Mongolian National University of Medical Sciences (No.: 2020/3–02).

## 3. Results

### 3.1. Clinical Data and Biochemical Parameters

Of the 178 individuals questioned, 46% (82) were male, while 54% (96) were female. The clinical and biochemical characteristics of the individuals are presented in Table 1. In our study, there was no statistically significant difference in age distribution between the T2DM and the control group (*p* > 0.05). Furthermore, we observed significant differences in several metabolic parameters. Fasting plasma glucose levels were 12.61 ± 0.45 mmol/L in the T2DM group and 5.360 ± 0.08 mmol/L in the control group (*p* < 0.0001) (Table 1). The HbA1c levels were 9.284 ± 0.21% in the T2DM group and 5.348 ± 0.08% in the control group (*p* < 0.0001). Triglyceride levels averaged 2.480 ± 0.20 mmol/L in the T2DM group and 1.675 ± 0.20 mmol/L in the control group (*p* < 0.0072). Total cholesterol levels were 5.046 ± 0.13 mmol/L in the T2DM group compared to 4.884 ± 0.12 mmol/L in the control group. HDL levels were 1.241 ± 0.03 mmol/L in the T2DM group and 1.373 ± 0.03 mmol/L in the control group. LDL levels were 2.918 ± 0.06 mmol/L in the T2DM group and 2.888 ± 0.05 mmol/L in the control group. There were no statistically significant differences in total cholesterol, HDL, and LDL levels between the groups (Table 1).

**TABLE 1:** Clinical and biochemical characteristics of T2DM and control groups.

Characteristics	T2DM (*n* = 89)	Control (*n* = 89)	*p* value
Age (years)	51.10 ± 1.151	47.92 ± 1.46	0.0896
FPG (mmol/L)	12.61 ± 0.45	5.360 ± 0.08	0.0001**^∗^**
HbA1c (%)	9.284 ± 0.21	5.348 ± 0.08	0.0001**^∗^**
TC (mmol/L)	5.046 ± 0.13	4.884 ± 0.12	0.3787
TG (mmol/L)	2.480 ± 0.20	1.675 ± 0.20	0.0072**^∗^**
HDL (mmol/L)	1.241 ± 0.03	1.373 ± 0.03	0.051
LDL (mmol/L)	2.918 ± 0.06	2.888 ± 0.05	0.7307

*Note:* TG, triglyceride. Bold ^∗^ *p* value was calculated with an independent t‐test; significant differences were observed between groups.

Abbreviations: FPG, fasting plasma glucose; HDL, high‐density lipoprotein; LDL, low‐density lipoprotein; TC, total cholesterol.

^∗^ *p* value was calculated with independent *t*‐test; significant differences were observed between groups.

### 3.2. The Frequency of Genotype and Allele of *ABCC8* rs1799854 SNPs

The genotypes, alleles, and genetic patterns of the *ABCC8* gene rs1799854 polymorphism were analyzed in case compared controls. The homozygous CC genotype frequency of the *ABCC8* gene rs1799854 polymorphism was 73 (82%) in the control group and 53 (59.5%) in the T2DM group. The heterozygous CT genotype frequency was 6 (6.7%) in the control group and 9 (10.1%) in T2DM group. The homozygous TT genotype frequency was 10 (11.3%) in the control group and 27 (30.4%) in the T2DM group, respectively (Table 2).

**TABLE 2:** Analysis of genotype and allele frequencies of the *ABCC8* gene rs1799854 polymorphism between groups.

	Control *n* (%)	Case *n*	OR (95% CI)	*p* value
*Genotype*				
СС	73 (82)	53 (59.5)	1	
СТ	6 (6.7)	9 (10.1)	2.06 (0.69–6.15)	0.2710
ТТ	10 (11.3)	27 (30.4)	3.71 (1.65–8.33)	**0.0013**

*Dominant model*				
CC	73 (82)	53 (59.5)	1	
СT + TT	16 (18)	36 (40.5)	3.09 (1.55–6.16)	**0.0016**

*Recessive model*				
ТТ	10 (11.2)	27 (30.3)	1	
CС + СT	79 (88.8)	62 (69.7)	0.29 (0.13–0.64)	**0.0028**

*Allele*				
С	152 (85.3)	115 (64.6)	1	
Т	26 (14.7)	63 (35.4)	3.2 (1.9–5.37)	**< 0.0001**

*Note:* Variables are expressed as actual numbers (*n*). Statistical significance was considered to be probable when *p* < 0.05. We used dominant and recessive genetic models to do the analysis. We found the odds ratios (ORs), their 95% confidence intervals (CIs), and the corresponding *p* values. The significance level for statistical tests was set at 0.05. Bold ^∗^ *p* value was calculated with an independent t‐test; significant differences were observed between groups.

There was a statistically significant difference between the two study groups (*p* < 0.05). The CT and TT genotypes of the *ABCC8* gene rs1799854 polymorphism were more common in the group with T2DM or *T* allele carriers, whereas the CC genotype was more common in the control group.

When calculating the allele prevalence of the polymorphism using the Hardy–Weinberg law, the *T* allele frequency was 89 (25%) and the C allele frequency was 267 (75%).

The C allele was detected in 152 (85.3%) of the control group and 115 (64.6%) of the case group. The mutant *T* allele was found in 63 (35.4%) of the cases and 26 (14.7%) of the controls. The control group showed an increased number of the C allele, whereas the case group showed the mutant *T* allele more frequently. A statistically significant difference was found between the *T* allele and the case group (*p* < 0.0001) (Table 2).

In the dominant genotype model, individuals without the mutant *T* allele occurred in 73 (82%) of the control group and 53 (59.5%) of the case group, while individuals with the *T* allele occurred in 16 (18%) of the control group and 36 (40.5%) of the case group. In the recessive model, individuals with the recessive C allele were 79 (88.8%) of the control group and 62 (69.7%) of the case group, while individuals without the recessive C allele were 10 (11.2%) of the control group and 27 (30.3%) of the case group. The case group observed a higher frequency of individuals with the dominant genotype model. In the recessive genotype model, those without the recessive C allele exhibited an increased risk of having the disease in the case group (*p* < 0.05) (Table 2).

### 3.3. The Frequency of Genotype and Allele of *ABCC8 rs1801261* SNPs

The genotypes, alleles, and genetic patterns of the *ABCC8* gene rs1801261 polymorphism were analyzed in case compared controls. The homozygous CC genotype frequency of the *ABCC8* gene rs1801261 polymorphism was 21 (23.6%) in the healthy group and 18 (20.2%) in the T2DM group. The frequency of the heterozygous CT genotype was 68 (76.4%) in the control group and 67 (75.3%) in the T2DM group. The homozygous TT genotype was absent in the control group and present in 4 individuals (4.5%) within the T2DM group. No statistically significant difference was seen between the two study groups (*p* > 0.05). The C allele was identified in 110 (61.8%) of the controls and 103 (59.2%) of the cases, whereas the mutant *T* allele was found in 71 (40.8%) of the cases with T2DM and 68 (38.2%) of the controls. The case group showed no statistically significant differences between the risk allele and nonrisk allele categories. However, because there were no individuals in the control group with the TT genotype, no statistically significant differences were found between the CC genotype and the CT genotype (Table 3).

**TABLE 3:** Analysis of genotype and allele frequencies of the *ABCC8* gene *rs1801261* polymorphism between groups.

	Control *n* (%)	Case *n* (%)	OR (95% CI)	*p* value
*Genotype*				
СС	21 (23.6)	18 (20.2)	1	
СТ	68 (76.4)	67 (75.3)	0.86 (0.42–1.77)	0.7201
ТТ	—	4 (4.5)	0.095 (0.004–1.8)	0.1078

*Dominant model*				
CC	21 (23.5)	18 (20.3)	1	
СT + TT	68 (76.5)	71 (79.7)	1.21 (0.59–2.48)	0.7174

*Recessive model*				
ТТ	85 (95.5)	89 (100)	1	
CС + СT	4 (0.5)	—	9.42 (0.49–177.8)	0.1208

*Allele*				
С	110 (61.8)	103 (59.2)	1	
Т	68 (38.2)	71 (40.8)	1.11 (0.72–1.71)	0.663

*Note:* Variables are expressed as actual numbers (*n*). Statistical significance was considered to be significant at *p* < 0.05.

HWE was assessed in the control group for the investigated polymorphisms. The genotype distribution of the *ABCC8* rs1799854 polymorphism showed a significant deviation from HWE (*χ* ^2^ = 47.40, *p* < 0.001). Similarly, the genotype distribution of *rs1801261* also significantly deviated from HWE (*χ* ^2^ = 34.01, *p* = *p* < 0.001). The observed and HWE‐expected genotype frequencies are presented in Table 4.

**TABLE 4:** Genotype distribution and Hardy–Weinberg equilibrium analysis of *ABCC8* polymorphisms in the control group.

SNP	Genotype	Observed *n* (%)	Expected *n* (%)	HWE *χ* ^2^	*p* value
rs1799854	CC	73 (82.0)	64.90 (72.9)		
	CT	6 (6.7)	22.20 (24.9)	47.40	*p* < 0.001
	TT	10 (11.3)	1.90 (2.2)		

rs1801261	CC	21 (23.5)	33.99 (38.2)		
	CT	68 (76.5)	42.02 (47.2)	34.01	*p* < 0.001
	TT	0 (0.0)	12.99 (14.6)		

## 4. Discussion

The genotypes of the rs1799854 polymorphism in the *ABCC8* gene differed between individuals with T2DM and those without a diabetes diagnosis. The *T* risk allele was more prevalent among individuals with T2DM. Our findings agreed with those of previous studies. The frequency of the mutant *T* allele was significantly higher in the case group than that in the control group (35.4%; *p* < 0.0001). This matched the findings of research in the United States (*p* = 0.04) [10] and the United Kingdom (*p* = 0.0001) and in the Japanese population (*p* < 0.007) [11]. Both recessive (OR = 0.29, *p* = 0.0028) and dominant (OR = 3.09, *p* = 0.0016) genetic models were significantly associated with T2DM in the present study. These findings are consistent with those reported in a Nigerian population [12]. However, previous studies have shown population‐specific differences, with the recessive model being associated with T2D in a Turkish population [13], whereas the dominant model was reported as a risk factor in a Japanese population (OR = 1.32, *p* = 0.004) [11]. This study found that the rs1799854 polymorphism in the *ABCC8* gene was not associated with Type 2 diabetes in South India, which is inconsistent with our results [14]. Wang et al.’s study in Han Chinese also found that this polymorphism was not associated with Type 2 diabetes [15]. The absence of association between this polymorphism and T2DM in a study conducted in Mexico contradicts our findings [16]. The rs1799854 polymorphism of the *ABCC8* gene exhibited a significant association with Type 2 diabetes in Japan (*p* = 0.0073), as reported by Norihida et al. This study indicated that the OR1.27 predicts the TT genotype of the rs1799854 polymorphism in the *ABCC8* gene increases susceptibility to Type 2 diabetes. This matches our study’s findings, as the TT genotype was more frequently detected in the case group [11]. A study by Marzana et al. indicates that this polymorphism partially influences the association between acute hyperglycemia and cardiovascular risk factor in Type 2 diabetes [17]. A study by Yang et al. in China showed that the rs1799854 polymorphism in the *ABCC8* gene may contribute to the development of Type 2 diabetes [7]. The rs1799854 variant of the *ABCC8* gene had no significant influence on response to SFUs treatment [18]. There is a point mutation in the *ABCC8* gene in the Solo population, with a wild‐type genotype of 30%, a mutant homozygote of 40%, and a mutant heterozygote of 30% [19]. The *T* allele and TT genotype in the −3 C/T polymorphism of *ABCC8* may provide a protective effect for the development of diabetic retinopathy (DR) (*p* = 0.036 for the TT genotype; *p* = 0.034 for the *T* allele) and proliferative DR (*p* = 0.042 and 0.025 for the TT genotype). Thus, the *T* allele and TT genotype at the −3 C/T polymorphism in the *ABCC8* gene may serve as a protective factor against the development of DR and proliferative diabetic retinopathy (PDR) [5]. The *T* allele and the CT and TT genotypes of the rs1799854 variant in the *ABCC8* gene were significantly associated with the risk of developing Type 2 diabetes, indicating their potential as effective genetic markers in Type 2 diabetes research across both combined populations of the Brazilian Amazon and native populations [20]. Overall, the findings of the present study support a significant association between the rs1799854 polymorphism of the *ABCC8* gene and susceptibility to T2DM. Specifically, the *T* allele and TT genotype were more frequently observed among individuals with T2DM, suggesting that this variant may contribute to disease risk. These results are consistent with findings reported in several populations, including those from the United States, the United Kingdom, Japan, Nigeria, China, and the Brazilian Amazon, which have also identified the rs1799854 polymorphism as a genetic factor associated with T2DM susceptibility. Furthermore, both dominant and recessive genetic models showed significant associations in the current study although previous studies have reported population‐specific differences regarding the most relevant inheritance model.

In contrast, studies conducted in South India, Han Chinese, and Mexican populations did not detect a significant association between rs1799854 and T2DM, highlighting inconsistencies across ethnic groups. These discrepancies may be explained by differences in genetic background, allele frequencies, linkage disequilibrium patterns, environmental exposures, lifestyle factors, sample size, and study design. In addition to its association with T2DM, the rs1799854 polymorphism has been implicated in other diabetes‐related outcomes, including cardiovascular risk and DR, suggesting a broader role in the pathophysiology of diabetes. Taken together, the available evidence indicates that the effect of the rs1799854 polymorphism may vary among populations, but the present findings provide further support for its potential role as a genetic marker of T2DM susceptibility. Future large‐scale studies involving diverse ethnic populations are needed to clarify the underlying mechanisms and confirm their clinical significance. Therefore, the observed association in the Mongolian population may reflect population‐specific genetic susceptibility that warrants further investigation in larger cohorts.

Studies by Radha et al. in India [14], Wang et al. among Han Chinese (2009) [6], and Rissanen et al. in a Finnish population (2000) [21] found no association between the rs1801261 polymorphism of the *ABCC8* gene and T2DM, confirming the findings of our study. The association with T2DM in studies conducted among Northern European Caucasians [10] and among French‐ Canadian Caucasians by Weisnagel et al. [22] is inconsistent with our findings. Also, the *ABCC8* gene rs1801261 polymorphisms were not associated with Type 2 diabetes in this study. But results suggested that the *ABCC8* rs1801261 polymorphism might influence T2DM susceptibility [23]. The study observed significant difference in T2D risk allele frequencies between the Mongolian population and the 1000G Caucasian population for several SNPs, including rs6723108 (TMEM163), whose risk allele approaches fixation in the Mongolian sample [24]. More research into the genomic architecture of these variations in relation to T2D susceptibility is needed to understand their significance across heterogeneous populations.

Ethnic variation may represent an important factor contributing to the inconsistent findings reported among different studies. Previous studies have demonstrated that susceptibility to T2D varies substantially among ethnic groups due to differences in genetic architecture, environmental influences, dietary habits, and lifestyle factors. The Mongolian population possesses a unique genetic background that may affect the contribution of *ABCC8* variants to disease susceptibility. Therefore, ethnic‐specific genetic effects should be considered when interpreting associations between *ABCC8* polymorphisms and T2D.

### 4.1. Limitations and Future Directions

This study did not consider environmental factors, lifestyle, or drugs that may also influence biochemical profiles. The low frequency of heterozygous genotypes for the *ABCC8* rs1799854 polymorphism is one of the study’s limitations. Although an analysis of the genotype distribution showed that it followed HWE, small deviations cannot be completely excluded. Future research may investigate the significance of these genotypes in various populations and evaluate the relationships between genetics and T2DM. Furthermore, investigating additional SNPs in the *ABCC8* gene may enhance our understanding of how these loci influence diabetes risk in the population at large.

## 5. Conclusions

The rs1801261 polymorphisms of the *ABCC8* gene were not associated with Type 2 diabetes. However, findings indicated that the *ABCC8* 1,799,854 polymorphism may affect susceptibility to T2DM in this study. Further genetic association studies involving *ABCC8* SNPs and T2DM will be required in a larger Mongolian population to investigate the genetic variables that contribute to the mechanisms of insulin insufficiency in T2DM.

## Funding

This study was supported by the postdoctoral grant of the Division for Science and Technology at the Mongolian National University of Medical Sciences.

## Conflicts of Interest

The authors declare no conflicts of interest.

## Data Availability

The data that support the findings of this study are available from the corresponding author upon reasonable request.
